# Clinical characteristics and impacts of traditional Chinese medicine treatment on the convalescents of COVID-19

**DOI:** 10.7150/ijms.52664

**Published:** 2021-01-01

**Authors:** Ya-Wen An, Bo Yuan, Jian-Chun Wang, Cheng Wang, Ting-Ting Liu, Shuo Song, Han-Qing Liu

**Affiliations:** 1 Central laboratory, Shenzhen Samii Medical Center, Shenzhen, Guangdong Province, 518118, China.; 2 Department of neurology, Shenzhen Samii Medical Center, Shenzhen, Guangdong Province, 518118, China.; 3 Department of Traditional Chinese Medicine, Shenzhen Samii Medical Center, Shenzhen, Guangdong Province, 518118, China.

**Keywords:** COVID-19, traditional Chinese medicine, discharged patients, laboratory examination

## Abstract

**Objectives:** A significant proportion of discharged COVID-19 patients still have some symptoms. Traditional Chinese medicine (TCM) has played an important role in the treatment of COVID-19, but whether it is helpful for discharged patients is still unknown. The aim of this study was to retrospectively analyze the impacts of TCM treatment on the convalescents of COVID-19.

**Methods:** A total of 372 COVID-19 convalescents from February 21 to May 3 in Shenzhen, China were retrospectively analyzed, 291 of them accepted clinically examined at least once and 191 convalescents accepted TCM.

**Results:** After retrospective analysis of the clinical data of convalescents accepted TCM treatment or not, we found that the white blood cell count, as well as serum interleukin-6 and procalcitonin decreased in TCM group. Serum γ-glutamyl transpeptidase was significantly decreased, while prealbumin and albumin increased in TCM group. Red blood cell, hemoglobin, and platelet count increased in TCM group. The mechanisms of TCM treatment might be the overall regulations, including balanced immune response, improved hematopoiesis and coagulation systems, enhanced functions of liver and heart, increased nutrient intake and lipid metabolism.

**Conclusions:** This study suggested that TCM treatment would be beneficial for discharged COVID-19 patients. However, long-term medical observation and further study with randomized trial should be done to confirm this result. Besides, the potential molecular mechanisms of TCM treatment should be further revealed.

## Introduction

The novel coronavirus disease (COVID-19) has lasted for more than 10 months since it was discovered in early 2020. There are more than 55 million laboratory confirmed COVID-19 patients worldwide and over 38 million of them have been discharged from hospital. The clinical characteristics of COVID-19 patients during hospitalization included myocardial injury 1, liver dysfunction 2, kidney related complications 3, abnormal peripheral blood system 4, hypolipidemia 5, and hematological complications 6. There is still no specific treatment available for COVID-19 patients currently, and the vaccines and targeted drugs are still in developing with time-consuming and arduous 7. Fortunately, many potential therapies including supportive interventions, immunomodulators, antiviral therapy, convalescent plasma transfusion, and traditional Chinese medicine (TCM), have been widely applied and have shown promising results 8.

In the absence of effective drugs and vaccines for severe acute respiratory syndrome coronavirus 2 (SARS-CoV-2) infectious, TCM has been proved as an effective treatment strategy to prevent and control the epidemic, especially in preventing mild symptoms from turning into severe ones 9. TCM has been recommended and widely used, and made vast contribution to the prevention, treatment and rehabilitation of COVID-19 in China since January 25, 2020 10. A total of 60,107 patients infected with SARS-CoV-2 in China were treated with TCM by February 17, 2020 11. A review described the herbal medicine and pattern identification for treating COVID-19, there were 8 pattern identifications and 23 herbal formulae for the mild stage, 11 pattern identifications and 31 herbal formulae for the moderate stage, 8 pattern identifications and 21 herbal formulae for the severe stage, and 6 pattern identifications and 23 herbal formulae for the recovery stage in the Chinese guidelines 12. The functions and mechanisms of TCM are very complex, and the most important rules may be to regulate and enhance immune system 13, and accelerate the rehabilitation. In addition, network pharmacology analysis predicted that TCM have general roles, including regulating viral infection, immune/inflammation reactions, and the hypoxia response *in vivo* 14.

The discharge criteria for COVID-19 patients were two negative SARS-CoV-2 virus tests and obvious improvement of pulmonary imaging symptoms. Despite meeting the discharge criteria, a significant proportion of discharged COVID-19 patients still had some symptoms including psychiatric symptoms, gastrointestinal reaction and low immunity 15, 16; some of them were even found to be recurrence of positive SARS‑CoV‑2 viral RNA one or more times 17. Therefore, dynamic surveillance of physical conditions such as peripheral blood system, liver function, kidney function and cardiovascular system, and how to promote recovery of discharged COVID-19 patients from the symptoms are very meaningful 18. The aims of the present study was to retrospectively analyze clinical characteristics of discharged COVID-19 patients in Shenzhen, China, calculate the proportion who used TCM, analyze the effectiveness of TCM for recovery period, and preliminarily reveal its underlying mechanisms.

## Methods

### Patients

Since the outbreak of the epidemic, the Shenzhen municipal government has taken a series of effective measures to control the epidemic. On February 21, Shenzhen was the first city in China to implement fixed-point isolation observation for discharged patients, and Shenzhen Samii Medical Center (SSMC, also called the Fourth people's Hospital of Shenzhen) was designated as the fixed-point isolation hospital for isolation and medical observation of discharged COVID-19 patients. Furthermore, the Health Commission of Shenzhen Municipality united with Beijing University of traditional Chinese medicine Shenzhen hospital, began to conduct comprehensive intervention of TCM during the convalescent of COVID-19 patients. More than three herbal formulas are recommended for rehabilitation of recovering cases, such as *Shen ling bai zhu san* 19, *Xiao chai hu tang* 20 and personalized treatments. These formulae could be presumably as rehabilitative measure for the convalescents of COVID-19 at SSMC. SSMC has received a total of 372 COVID-19 convalescents from February 21 to May 3, 2020. Among these, 291 convalescents were clinically examined from March 4 to May 3, and 191 convalescents accepted TCM decoction, 158 of them accepted at least once blood tests before or after taking TCM decoction from March 5 to May 3, 2020.

### Blood routine and biochemical indices

Blood tests data of convalescents were retrospectively analyzed. Sample collection and test protocols were consistent as descried 15. In brief, a total of 3 mL blood was extracted from every patient, and the serum was separated by centrifugation at 2327 × *g* for 15 min (L550; Hunan xiangyi laboratory instrument development co. LTD, China). Routine blood tests were performed using an automatic blood cell analyzer (XN1000; Sysmex, Japan). Serum biochemistry was performed using an automated biochemical analyzer (Cobas C501; Roche, Germany) after 30 min at 56 °C for virus inactivation. Interleukin-6 (IL-6) level was determined using an automatic chemiluminescence immunoassay system (Caris 200; WANTAI BioPharm, China). The reagents, controls, and calibrators used were original and operated according to the manufacturer's instructions.

### Ethical statement

This study was approved by the Ethics Committee of Shenzhen Samii Medical Center (SSMC-R-20200401) and written informed consent was obtained from each patient.

### Statistics

All analyses were conducted with SPSS software version 19.0. Continuous variables were expressed as the means ± standard deviation and a one-way analysis of variance (Tamhane's T2) was used to compare inter-group differences. *P* < 0.05 was an indication of statistical significance.

## Results

### Influences of TCM treatment on immune responses of discharged COVID-19 patients

Routine blood indices included white blood cell (WBC), neutrophil (NEUT), lymphocyte (LYM), monocyte (MONO), eosinophil (EOS), and basophilic granulocyte (BASO) count. As shown in **Table 1,** the WBC (5.68 *vs* 6.01 ×10^9^/L), LYM (1.73* vs* 1.84 ×10^9^/L) and MON (0.44* vs* 0.49 ×10^9^/L) in TCM group were significantly decreased than non-treatment group (*P* < 0.01, *P* < 0.05).

According to inflammatory status with SARS-CoV-2 infection, IL-6 is the most important marker of immune factors. The IL-6 level of TCM group was dramatically lower than the non-treatment group (4.47 *vs* 18.31 pg/mL, *P* < 0.05). Procalcitonin (PCT^1^) is another important index of infection disease, and the PCT^1^ level in TCM group was significantly lower than the non-treatment group (0.02 *vs* 0.04 ng/mL, *P* < 0.05). However, the level of hypersensitive-C reaction protein (hs-CRP) and serum amyloid A (SAA) showed no significant differences between the two groups (**Table 1**).

### Influences of TCM treatment on the functions of liver, kidney, and cardiovascular systems

It was reported that coronavirus affected lung, liver, kidney, and cardiovascular function, but the criteria used for the discharged COVID-19 patients were only based on a negative viral nucleic acid test and improvement of pulmonary infection symptoms. To investigate the functions of liver, kidney and cardiovascular system of these discharged patients, we retrospectively analyzed the blood and serum biochemistry data which included hepatic and renal function indices, blood lipid levels, as well as myocardial enzyme spectrums.

After comparison of serum indices of hepatic function, it was found that serum γ-glutamyl transpeptidase (GGT) was significantly reduced after TCM treatment (27.31 *vs* 35.10 U/L, *P* < 0.01, **Table 2**). This suggested a protective effect on hepatocytes. Prealbumin (PA) in serum was significantly improved in TCM group (0.35 *vs* 0.29 g/L, *P* < 0.05, **Table 2**). This index also predicted improvement of recovery and nutrition uptake enhancement.

The renal function indices between TCM treatment and non-treatment groups showed no significant differences (**Table 2**).

Blood lipid level results showed that the serum value of apolipoprotein B (ApoB, 0.93 *vs* 0.85 g/L) and lipoprotein C (LDL-C, 3.13 *vs* 2.91 mmol/L) of the TCM group were increased compared to the non-treatment group (*P* < 0.001, *P* < 0.05, **Table 2**). Besides, the LDL-C level in TCM group was a little higher than the reference range, this also should be noticed. These results suggested after TCM treatment, the blood lipid level should be monitored during the follow-up clinical examination.

After contrasting the myocardial enzyme spectrums between groups with TCM treatment or not, lactic dehydrogenase (LDH) was decreased in the TCM group compared to the non-treatment group (166.32 *vs* 174.70 U/L). This might reflect the cardioprotective effect of TCM treatment.

These indices showed significant differences between the two groups, and were closed relating to the liver function and cardiovascular system. All these data indicated that TCM might regulate lipid metabolism and further influence liver and cardiovascular system.

### Influences of TCM treatment on hematopoiesis and coagulation of convalescents from COVID-19

As a recent research indicated, low platelet count is associated with increased risk of severe disease and mortality in patients with COVID-19 21, and thus should serve as clinical indicator of worsening illness during hospitalization and early recovery stage. Blood routine indices indicated that the hematopoiesis and coagulation, several related indices were dramatically changed after TCM treatment (**Table 3**), including platelet count, erythrocyte and hemoglobin parameters. The elevated platelet count (PLT, 238.62 *vs* 191.36 ×10^9^/L, *P* < 0.001) and plateletocrit (PCT^2^, 0.25% *vs* 0.21%, *P* < 0.001) indicated the TCM treatment could promote the recovery of clotting function. The red blood cell (RBC, 4.66 *vs* 4.47 ×10^12^/L, *P* < 0.001) count and hematocrit (HCT, 42.70% *vs* 41.36%, *P* < 0.01) were also increased after TCM treatment, which might suggest TCM could promote erythropoiesis. Hemoglobin (HGB, 139.64 *vs* 132.97 g/L, *P* < 0.001) and mean corpuscular-hemoglobin concentration (MCHC, 326.58 *vs* 321.20 g/L, *P* < 0.0 1) content were significantly increase after TCM treatment, which further proved the TCM supplementary treatment was beneficial to the recovery from COVID-19.

## Discussion

More than 38 million of COVID-19 patients have been discharged from hospitals worldwide. Our previous study found that despite meeting discharge criteria, some discharged COVID-19 patients still had some symptoms, such as depression, anxiety, fatigue, loss of appetite, abnormal blood parameters, etc., 15-18, 22. TCM might play roles of antivirus, anti-inflammation and immunoregulation, and target organs protection in the management of COVID-19 23. Several herbal formulae are recommended for rehabilitation of recovering cases 24. The present retrospective analysis confirmed that TCM treatment during the convalescences of COVID-19 was beneficial to the discharged patients who were still with some symptoms.

Peripheral blood cell distribution characteristics could reflect the innate immune function, hematopoiesis, and coagulation of body directedly. Patients with COVID-19 have lower counts of leucocytes, lymphocytes, eosinophils, platelets, and hemoglobin 4. A treatment efficacy analysis for COVID-19 found that, comparing the hemograms between admission and discharge of hospital, the numbers of erythrocytes, hemoglobin concentration decreased when the patients discharged 25, this might be caused by the therapeutic medicines for antivirus. An empirical study from Wuhan, Hubei province, China proved that the levels WBC, EOS %, NEUT#, LYM#, EOS#, BASO#, PLT and PCT increased, while the levels of RBC, HGB, HCT, and MPV decreased in COVID-19 patients when they discharged comparing with admission. And TCM treatment significantly ameliorated the immune ability against SARS-CoV-2 in patients via reversing the levels of these indices 25. Anemia was proved to be associated with severe COVID-19) infection 26, and decreased HGB is common in forms of anemia. MCHC is lower in small cell anemia. Our results showed that, during the convalescences period, TCM treatment could elevate the RBC (4.66 *vs* 4.47 ×10^12^/L) compared with non-treatment group, HGB (139.64 *vs* 132.97 g/L), as well as MCHC (326.58 *vs* 321.20 g/L) which suggested that TCM treatment was improved the production of RBC and increased the HGB concentration. This may be beneficial to the patient's recovery.

Increased cytokine levels (IL-6, IL-10, and TNF-α) are associated with severe COVID-19 27. IL-6 was secreted by monocytes, macrophages, and dendritic cells when they were activated by beta coronavirus infected, and elevated serum IL-6 correlates with respiratory failure 28. The inhibitory effect of TCM on cytokines in COVID-19 patients has been demonstrated already. For example, *Lian hua qing wen* significantly inhibited SARS-CoV-2 replication in Vero E6 cells and markedly reduced pro-inflammatory cytokines including IL-6 13. By monitoring the expression of IL-6 in serum during rehabilitation, we found that the level of IL-6 was significantly reduced after TCM treatment, suggesting that the level of inflammation in the body improved.

With the outbreak of COVID-19, cases of liver damage or dysfunction have been reported among patients with COVID-19 29. This might be caused by viral infection or the use of potentially hepatotoxic drugs. In a previous study, we found that a significant proportion of discharged COVID-19 patients with abnormal liver function indicators even after 2 months of follow-up 18. In the present study, serum aspartate aminotransferase (AST) level of convalescents was back to normal. Gamma-glutamyl transferase (GGT), a diagnostic biomarker for cholangiocyte injury, was elevated in 30 (54%) of 56 patients with COVID-19 during hospitalization 30. The elevated PA and ALB levels indicated TCM treatment could improve metabolic function of liver cells, which further increase nutrient absorption. ApoB could carry triglycerides and cholesterol in plasma. Elevated ApoB and LDL-C in the TCM group might be caused by the increased nutrients intake and improved metabolism of liver. The toxicity of TCM is often concerned, especially liver toxicity 31. However, the present study suggested that TCM is safe for convalescent COVID-19 patients and even has some protective effects on the liver.

Although the respiratory and immune systems are the major targets of COVID-19, acute kidney injury and proteinuria have also been observed. In addition to the direct virulence of SARS-CoV-2, factors contributing to acute kidney injury included systemic hypoxia, abnormal coagulation, and possible drug or hyperventilation-relevant rhabdomyolysis 32. Previously research reported that renal abnormalities such as proteinuria and hematuria often resolved in COVID-19 patients within 3 weeks after the onset of symptoms 3. Anti-COVID-19 medicines have been reported to cause adverse side effects to kidney. Some TCM including *Huang Qi, Fu Ling, Bai Zhu, Di Huang, Shan Yao, etc.,* might be beneficial in treating renal injury in the battle of COVID-19. The present study showed that TCM treatment didn't significantly affect the recovery of blood renal indicators, which might due to the fact that renal indicators have all returned to normal after discharge. In the future study, we will detect cytokines in urine to further reveal the recovery of renal function of COVID-19 patients and the influence of TCM.

In conclusion, TCM treatment could significantly promote the recovery of COVID-19 patients in the convalescent stage and its mechanism might be the overall regulations on body, including balanced immune response, enhanced liver function, increased nutrient intake and lipid metabolism.

However, there are some limitations in the present study. First of all, this study was a retrospective analysis, have many bias or confounder variables not been adjusted. For example, the monocyte and IL-6 in convalescence period are naturally decreased, and may bring bias. In addition, the sample size was small and the follow-up time was short. Besides, the potential mechanisms of TCM treatment and effective compounds about TCM formula are still unclear. Above all, further study with prospective design or randomized trial should be done to confirm these results, and the molecular mechanisms of TCM treatment should be the further research directions.

## Figures and Tables

**Table 1:** Influences of TCM treatment on immune factors in COVID-19 convalescents

Indices (unit)	Reference range	Non-treatment (n = 425)	TCM treatment (n = 143)	*P* value
WBC count (10^9^/L)	3.5-9.5	6.01±1.38	5.68±1.05**↓	0.0078
LYM count (10^9^/L)	1.1-3.2	1.84±0.55	1.73±0.43*↓	0.0234
MON count (10^9^/L)	0.1-0.6	0.49±0.17	0.44±0.12**↓	0.0033
NEUT count (10^9^/L)	1.8-6.3	3.52±1.17	3.32±0.90	0.0734
EOS count (10^9^/L)	0.02-0.52	0.13±0.09	0.15±0.14	0.1286
BASO count (10^9^/L)	0-0.06	0.04±0.02	0.04±0.01	0.8098
IL-6 (pg/mL)	<7	18.31±54.06	4.47±17.53*↓	0.0113
Hs-CRP (mg/dL)	0-0.5	0.42±0.69	0.58±0.64	0.080
SAA (mg/L)	0-10	6.20±5.51	6.06±8.82	0.8293
PCT^1^ (ng/mL)	0-0.5	0.04±0.04	0.02±0.03*↓	0.0372

Continuous variables were analyzed using one-way ANOVA (Tamhane's T2) test. *P<0.05, **P<0.01 *vs* non-treatment group. The symbols ↓ and ↑ represented significantly higher or lower.

**Table 2:** Comparison of blood biochemistry indices between different treatments in COVID-19 convalescents

Indices	Reference range	Non-treatment(n = 425)	TCM treatment(n = 143)	*P* value
**Liver function**				
GLB (g/L)	20-40	26.81±4.66	26.61±3.83	0.6427
A/G	1.5-2.5	1.75±0.31	1.79±0.30	0.1587
AST/ALT	0.5-1.5	1.42±0.64	1.36±0.63	0.3558
GGT (U/L)	7-60	35.10±33.06	27.31±18.13**↓	0.0088
ALT (U/L)	7-50	20.70±19.87	20.55±16.36	0.9398
TBA (µmol/L)	0-10	7.09±6.74	7.82±4.54	0.2386
PA (g/L)	0.2-0.4	0.29±0.06	0.35±0.62*↑	0.0492
ALB (g/L)	35-52	45.68±4.11	46.66±2.85**↑	0.0098
TP (g/L)	65-85	72.50±4.71	73.27±3.89	0.0818
**Kidney function**				
CysC (mg/L)	0-1.5	0.96±0.22	0.96±0.15	0.8490
UA (µmol/L)	119-416.5	315.30±78.2	316.80±76.43	0.8452
Urea (mmol/L)	2.78-8.07	4.39±1.40	4.35±1.05	0.7672
Cr (µmol/L)	45-104	58.49±17.54	58.60±16.30	0.9451
CO2CP (mmol/L)	21-31	24.54±2.79	24.79±2.38	0.3435
β2-MG (mg/L)	0.8-2.2	1.768±0.68	1.69±0.47	0.2247
**Blood lipid level**				
ApoAⅠ/ApoB	0.8-2.2	1.80±0.58	1.70±0.54	0.0799
LP(a) (mg/dL)	0-30	20.76±22.76	19.02±21.99	0.4322
ApoB (g/L)	0.60-1.33	0.85±0.23	0.93±0.26***↑	0.0009
ApoAⅠ (g/L)	1.04-2.25	1.44±0.28	1.46±0.23	0.4438
TC (mmol/L)	0-5.2	4.98±1.15	5.18±1.18	0.0749
TG (mmol/L)	0-2.6	2.42±2.02	2.59±1.47	0.3644
HDL-C (mmol/L)	1-1.55	1.20±0.35	1.17±0.29	0.3449
LDL-C (mmol/L)	1.90-3.10	2.91±0.93	3.13±1.05 *↑	0.0194
**Myocardial enzyme spectrums**				
α-HBDH (U/L)	78-182	125.46±25.51	124.07±26.12	0.5842
LDH (U/L)	135-214	174.70±37.35	166.32±32.89*↓	0.0195
CK-MB (U/L)	0-25	6.14±4.00	5.83±3.41	0.4233
CK (U/L)	20-200	26.26±39.13	20.18±13.97	0.0764
AST (U/L)	15-40	22.45±12.93	22.14±9.74	0.7984

Continuous variables were analyzed using one-way ANOVA (Tamhane's T2) test. *P<0.05, **P<0.01, ***P<0.001 *vs* non-treatment group. The symbols ↓ and ↑ represented significantly higher or lower.

**Table 3:** Comparison of blood routine indices between different treatments in COVID-19 convalescents

Indices (unit)	Reference range	Non-treatment (n = 425)	TCM treatment (n = 143)	*P* value
**Platelet system**				
PLT (10^9^/L)	125-350	191.36±105.01	238.62±56.99***↑	0.0000
PCT^2^ (%)	0.18-0.39	0.21±0.10	0.25±0.05***↑	0.0000
MPV (fL)	6.5-12	10.92±1.10	10.72±0.94	0.0590
P-LCR (%)	17.5-42.3	32.07±8.65	30.60±7.70	0.0747
PDW	15-17	12.88±2.72	12.73±2.16	0.5506
**Erythrocyte system**				
RBC (10^12^/L)	3.8-5.1	4.47±0.58	4.66±0.45***↑	0.0003
MCV (fL)	82-100	93.04±5.75	91.78±3.88*↓	0.0145
RDW-SD (fL)	38.2-49.2	45.58±6.82	43.84±4.21**↓	0.0042
RDW-CV (%)	12.1-14.3	13. 41±1.56	13.04±1.12**↓	0.0089
HCT (%)	35-45	41.36±4.28	42.70±3.57**↑	0.0008
**Hemoglobin parameter**				
HGB (g/L)	115-150	132.97±15.77	139.64±14.41***↑	0.0000
MCH (pg)	27-34	29.86±1.80	29.97±1.62	0.5112
MCHC (g/L)	316-354	321.20±12.93	326.58±10.27***↑	0.0000

Continuous variables were analyzed using one-way ANOVA (Tamhane's T2) test. *P<0.05, **P<0.01, ***P<0.001 *vs* non-treatment group. The symbols ↓ and ↑ represented significantly higher or lower.
